# enChIP systems using different CRISPR orthologues and epitope tags

**DOI:** 10.1186/s13104-018-3262-4

**Published:** 2018-02-27

**Authors:** Toshitsugu Fujita, Miyuki Yuno, Hodaka Fujii

**Affiliations:** 1 Department of Biochemistry and Genome Biology, Hirosaki University Graduate School of Medicine, 5 Zaifu-cho, Hirosaki, Aomori 036-8562 Japan; 2 Chromatin Biochemistry Research Group, Combined Program on Microbiology and Immunology, Research Institute for Microbial Diseases, Osaka University, 3-1 Yamadaoka, Suita, Osaka 565-0871 Japan

**Keywords:** enChIP, Sa-dCas9, ChIP, Chromatin immunoprecipitation, CRISPR

## Abstract

**Objective:**

Previously, we developed the engineered DNA-binding molecule-mediated chromatin immunoprecipitation (enChIP) technology, which isolates specific genomic regions while preserving their molecular interactions. In enChIP, the locus of interest is tagged with engineered DNA-binding molecules such as the clustered regularly interspaced short palindromic repeats (CRISPR) system, consisting of a catalytically inactive form of Cas9 (dCas9) and guide RNA, followed by affinity purification of the tagged locus to allow identification of associated molecules. In our previous studies, we used a 3xFLAG-tagged CRISPR system from *Streptococcus pyogenes* (*S. pyogenes*). In this study, to increase the flexibility of enChIP, we used the CRISPR system from *Staphylococcus aureus* (*S. aureus*) along with different epitope tags.

**Results:**

We generated a plasmid expressing *S. aureus* dCas9 (Sa-dCas9) fused to a nuclear localization signal (NLS) and a 3xFLAG-tag (Sa-dCas9-3xFLAG). The yields of enChIP using Sa-dCas9-3xFLAG were comparable to those using *S. pyogenes* dCas9 fused with an NLS and a 3xFLAG-tag (3xFLAG-Sp-dCas9). We also generated another enChIP system using Sp-dCas9 fused with an NLS and a 2xAM-tag (Sp-dCas9-2xAM). We obtained high enChIP yields using this system as well. Our findings indicate that these tools will increase the flexibility of enChIP analysis.

## Introduction

Identification of the molecules associated with a genomic region of interest in vivo is an essential step in understanding the regulatory mechanisms underlying its functions. To this end, we previously developed engineered DNA-binding molecule-mediated chromatin immunoprecipitation (enChIP) technology to isolate genomic regions of interest while retaining their molecular interactions [1, 2]. In enChIP, engineered DNA-binding molecules such as transcription activator-like (TAL) proteins [3] or the clustered regularly interspaced short palindromic repeats (CRISPR) system [4, 5], which consists of a catalytically inactive form of Cas9 (dCas9) and guide RNA (gRNA), are used to tag the locus of interest, followed by affinity purification of the tagged locus to allow identification of associated molecules. For specific and efficient affinity purification, we usually use 3xFLAG-tag in conjunction with antibody (Ab) against the epitope tag. Locus-tagging can be done in the cell by expressing engineered DNA-binding molecules [1, 2, 6–9] or in vitro using recombinant or synthetic engineered DNA-binding molecules [10, 11]. Combination of enChIP with mass spectrometry (MS), RNA sequencing, and next-generation sequencing (NGS) has enabled us to identify proteins [1, 2, 6], RNAs [7], and genomic regions [9, 11] interacting with specific genomic regions of interest in a non-biased manner.

In our previous enChIP studies, we used the CRISPR system from *Streptococcus pyogenes* (*S. pyogenes*), the most extensively analyzed version of this system. However, because of its requirement for the protospacer adjacent motif (PAM) sequence (5′-NGG-3′), some DNA sequences cannot be targeted by this CRISPR system.

In addition to the *S. pyogenes* CRISPR system, those from other species have been used for genome editing or other purposes. Among others, the CRISPR system from *Staphylococcus aureus* (*S. aureus*) [12] has been used widely. Since the size of its Cas9 is smaller than that of the *S. pyogenes* Cas9, the length of its expression plasmid can be shorter than that of the *S. pyogenes* Cas9, enabling higher efficiency of transfection or transduction. In addition, since its PAM sequence (5′-NNGRRT-3′ or 5′-NNGRR(N)-3′) is distinct from the *S. pyogenes* system, DNA sequences difficult to be targeted by the *S. pyogenes* system can be targeted by the *S. aureus* system, increasing the flexibility of the enChIP technology.

To solve the potential problem of the *S. pyogenes* CRISPR system and increase the flexibility of enChIP analysis, we developed an enChIP system utilizing the 3xFLAG-tagged CRISPR system from *S. aureus*. In addition, we developed another enChIP system using the *S. pyogenes* CRISPR system fused to a different epitope tag. In combination, these tools might be used to constitute a sequential enChIP system with reduced background noise.

## Main text

### Materials and methods

#### Plasmids

3xFLAG-dCas9/pCMV-7.1 (Addgene #47948) was described previously [1]. To construct Sa-dCas9-NLS-3xFLAG/pcDNA3.1 (Addgene #98041), pcDNA3.1/myc-His(−) A (Invitrogen) was digested with *Nhe*I. After a blunting reaction, the plasmid was digested with *Not*I and treated with bacterial alkaline phosphatase (*E. coli* C75) (Takara Bio). The MSP2262 plasmid (Addgene #70703) [12] was digested with *Nco*I. After a blunting reaction, the plasmid was further digested with *Not*I. The cleaved pcDNA3.1/myc-His(−) A and the coding sequence of Sa-dCas9-3xFLAG from MSP2262 were purified by agarose gel electrophoresis and ligated.

To construct vectors for expression of gRNAs targeting the human *interferon regulatory factor*-*1* (*IRF*-*1*) locus, two oligonucleotides for each gRNA were annealed, phosphorylated, and inserted into *Bsm*BI-cleaved BPK2660 plasmid (Addgene #70709) [12] to yield hIRF-1 Sa gRNA #409 (Addgene #98134) and hIRF-1 Sa gRNA #351 (Addgene #105283). The nucleotide sequences were as follows: #409 (Addgene #98134), 5′-cacctctccagtgggaacactggg-3′, and 5′-aaaccccagtgttcccactggaga-3′; #351 (Addgene #105283), 5′-caccccagtgggatatcaagaagg-3′ and 5′-aaacccttcttgatatcccactgg-3′.

To construct pLenti_dCas9-2xAM (Addgene #92220), the coding region of Sp-dCas9 fused with an NLS and two copies of the AM-tag sequence (QDPQRKGNVILSQAY) was inserted into the CSII-U6-gRNA-CBh-3xFLAG-PA-dCas9-P2A-Puro plasmid (Addgene #83306) cleaved with *Bam*HI and *Age*I. To construct pLenti_dCas9-2xAM_hIRF-1 (Addgene #92221), two oligonucleotides (5′-caccgcgggggcgctgggctgtcc-3′ and 5′-aaacggacagcccagcgcccccgc-3′) were annealed, phosphorylated, and inserted into pLenti_dCas9-2xAM digested with *Bbs*I.

#### Cell lines

The 293T cell line was derived from the 293 cell line isolated human embryonic kidneys (HEK) and transformed with the SV40 large T antigen [13]. The HT1080 cell line was derived from human fibrosarcoma [14] and purchased from ATCC (CCL-121). The 293T cell line, HT1080 cell line, and HT1080 derivatives were maintained in DMEM (Wako) supplemented with 10% fetal calf serum (FCS).

#### Transfection of Sa-dCas9-3xFLAG and gRNA

For transient expression of Sa-dCas9-3xFLAG, 3 µg of Sa-dCas9-NLS-3xFLAG/pcDNA3.1 was transfected into 1 × 10^6^ 293T cells using Lipofectamine 3000 (Life Technologies). For transient expression of 3xFLAG-Sp-dCas9 or Sa-dCas9-3xFLAG and their gRNA targeting the *IRF*-*1* locus, 1.5 µg of 3xFLAG-Sp-dCas9/pCMV-7.1 or Sa-dCas9-NLS-3xFLAG/pcDNA3.1 in the absence or presence of 1.5 µg of the corresponding gRNA expression plasmid (gRNA-hIRF-1 #12 (Addgene #61079) for Sp-dCas9 or hIRF-1 gRNA #351 (Addgene #105283) and hIRF-1 gRNA #409 (Addgene #98134) for Sa-dCas9) was transfected into 1 × 10^6^ 293T cells using Lipofectamine 3000.

#### Transduction of pLenti_dCas9-2xAM and pLenti_dCas9-2xAM_hIRF-1

For transduction of pLenti_dCas9-2xAM and pLenti_dCas9-2xAM_hIRF-1, 5.1 µg of each plasmid was transfected into 1 × 10^6^ 293T cells along with pCAG-HIVgp (RIKEN BioResource Center RDB04394) and pCMV-VSV-G-RSV-Rev (RDB04393) [15] (3 µg each) using Lipofectamine 3000. Two days after transfection, viral supernatant was harvested and used for infection of HT1080 cells. Infected cells were selected in culture media containing puromycin (1 µg/ml).

#### Immunoblot analysis

Nuclear extracts (NE) were prepared using the NE-PER Nuclear and Cytoplasmic Extraction Reagents (Thermo Fisher Scientific). NE (10 µg) was subjected to immunoblot analysis with anti-FLAG M2 Ab (F1804, Sigma-Aldrich) or Ab against AM-tag (39715, Active Motif) as described previously [1].

#### enChIP-real-time PCR

enChIP-real-time PCR was performed as described previously [1]. Anti-FLAG M2 Ab (3 µg Ab/4 × 10^6^ cells) or Ab against AM-tag (2 µg Ab/4 × 10^6^ cells) were used. Primers used in the analysis are shown in Table 1.

**Table 1:** Primers used in this study

Number	Name	Sequence (5′ → 3′)	Experiments
27222	hSox2-prom-F	attggtcgctagaaacccatttatt	Real-time PCR in Figs. 1e and 2d (SOX2)
27223	hSox2-prom-R	ctgccttgacaactcctgatacttt	Real-time PCR in Figs. 1e and 2d (SOX2)
27310	hIRF1-prom-F	cgcctgcgttcgggagatatac	Real-time PCR in Figs. 1e and 2d (IRF1)
27312	hIRF1-prom-R1 + 2	ctgtcctctcactccgccttgt	Real-time PCR in Figs. 1e and 2d (IRF1)

### Results and discussion

#### enChIP analysis using the *S. aureus* CRISPR system

**Fig. 1:**
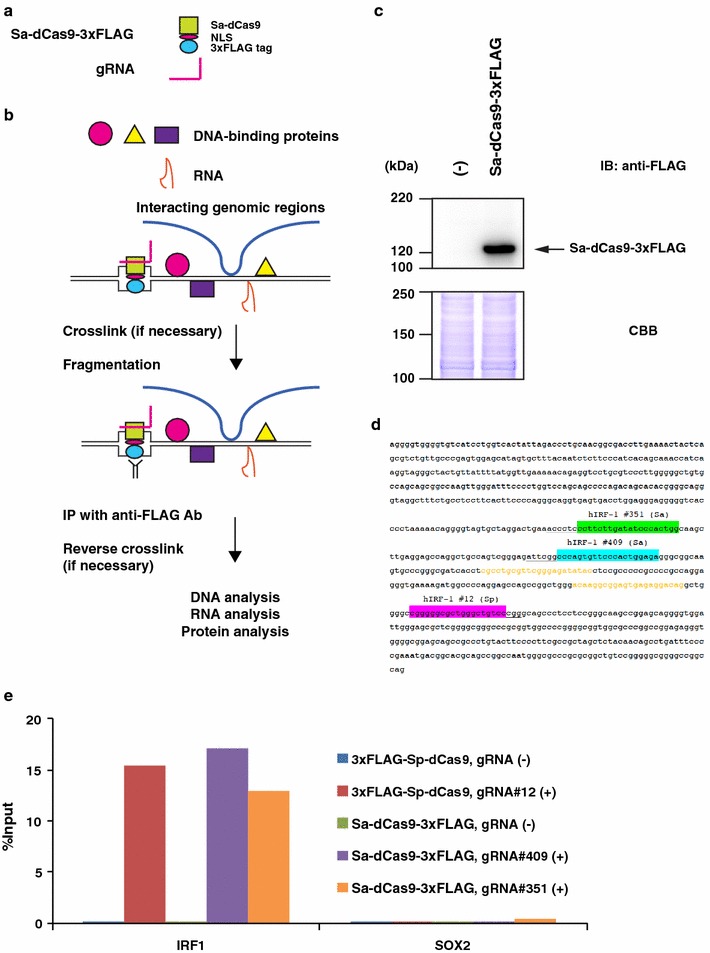
enChIP system using *S. aureus* CRISPR. **a** The *S. aureus* CRISPR system for enChIP. The system is composed of a fusion protein, Sa-dCas9-3xFLAG (consisting of Sa-dCas9, an NLS, and a 3xFLAG-tag) and a gRNA. **b** Scheme of the enChIP system using *S. aureus* CRISPR. The Sa-dCas9-3xFLAG and gRNA are expressed for locus-tagging in the target cells. The cells are crosslinked (if necessary), lysed, and fragmented by sonication or other methods. Chromatin complexes containing the CRISPR complex are immunoprecipitated with anti-FLAG Ab, and the crosslink (if used) is reversed. Molecules (DNA, RNA, proteins, etc.) associated with the target genomic region can be identified by downstream analyses (e.g., nucleic acids by next-generation sequencing, proteins by mass spectrometry). **c** Expression of Sa-dCas9-3xFLAG. Plasmid expressing Sa-dCas9-3xFLAG was transfected into 293T cells. Nuclear extracts were prepared and subjected to immunoblot analysis (IB) with anti-FLAG Ab. Coomassie Brilliant Blue (CBB) staining is shown as a protein loading control. **d** Positions of gRNAs in the *IRF*-*1* promoter. Green highlight: hIRF-1 #351 (Sa) gRNA; blue highlight: hIRF-1 #409 (Sa) gRNA; magenta highlight: hIRF-1 #12 (Sp) gRNA; underlines: PAM sequences; orange letters: primers for enChIP-PCR analysis. **e** Isolation of the *IRF*-*1* locus by enChIP using the *S. aureus* CRISPR system. Real-time PCR analysis of chromatin complexes isolated by enChIP is shown. An irrelevant locus (*SOX2*) was analysed as a negative control. The *S. pyogenes* CRISPR system was used as a positive control for enChIP

To determine whether the *S. aureus* CRISPR system could be used for enChIP analysis (Fig. 1a, b), we constructed a mammalian expression plasmid (Sa-dCas9-NLS-3xFLAG/pcDNA3.1) encoding Sa-dCas9 fused with an NLS and the 3xFLAG-tag (Sa-dCas9-3xFLAG), and transiently transfected it into 293T cells. Expression of Sa-dCas9-3xFLAG was confirmed by immunoblot analysis (Fig. 1c).

Next, for enChIP analysis, we transfected Sa-dCas9-NLS-3xFLAG/pcDNA3.1 alone or along with a plasmid expressing gRNA targeting the human *IRF*-*1* promoter (hIRF-1 gRNA #351 or hIRF-1 gRNA #409) (Fig. 1d). As a positive control, we also transfected 3xFLAG-Sp-dCas9/pCMV-7.1 expressing 3xFLAG-Sp-dCas9, alone or with a plasmid expressing gRNA targeting the human *IRF*-*1* promoter (gRNA-hIRF-1 #12) (Fig. 1d). Two days after transfection, cells were crosslinked with formaldehyde. Crosslinked chromatin was fragmented by sonication. Next, chromatin complexes containing Sa-dCas9-3xFLAG or 3xFLAG-Sp-dCas9 were immunoprecipitated with anti-FLAG Ab. For both Sp- and Sa-dCas9, real-time PCR showed that the *IRF*-*1* promoter region was specifically detected only in the immunoprecipitants prepared from 293T cells transfected with gRNA targeting the *IRF*-*1* promoter (Fig. 1e). The yields of enChIP for Sa-dCas9 were comparable with those for Sp-dCas9. These results showed that enChIP using the *S. aureus* CRISPR system can also specifically and efficiently isolate target genomic regions.

#### Generation of a lentiviral expression system for enChIP using *S. pyogenes* CRISPR and 2xAM-tag

**Fig. 2:**
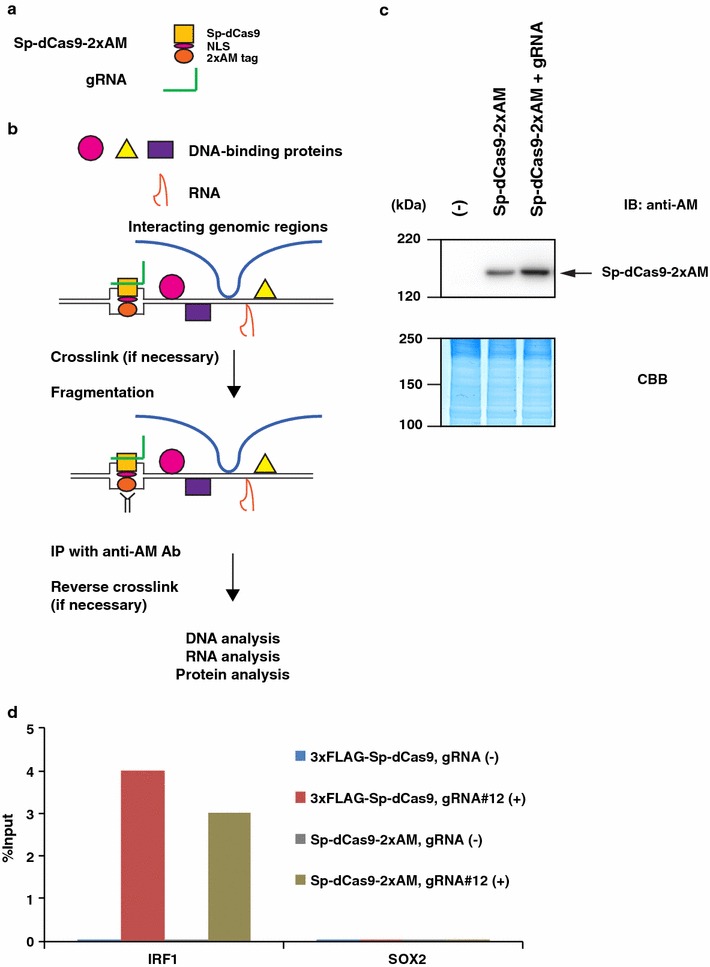
enChIP using the *S. pyogenes* CRISPR system tagged with the 2xAM-tag. **a** The *S. pyogenes* CRISPR system tagged with 2xAM-tag for enChIP. The system is composed of a fusion protein Sp-dCas9-2xAM (consisting of Sp-dCas9, an NLS, and a 2xAM-tag) and a gRNA. **b** Scheme of the enChIP system using *S. pyogenes* CRISPR tagged with the 2xAM-tag. After expression of Sp-dCas9-2xAM and a gRNA for locus-tagging in target cells, enChIP is performed using an Ab against the AM-tag, as shown in Fig. 1b. **c** Expression of Sp-dCas9-2xAM. pLenti_dCas9-2xAM or pLenti_dCas9-2xAM_hIRF-1 was transduced into HT1080 cells. After puromycin selection, expression of Sp-dCas9-2xAM was detected by immunoblot analysis with Ab against AM-tag. **d** Isolation of the *IRF*-*1* locus by the *S. pyogenes* CRISPR system tagged with 2xAM-tag. Real-time PCR analysis was performed on chromatin complexes isolated by enChIP. An irrelevant locus (*SOX2*) was analysed as a negative control. The *S. pyogenes* CRISPR system tagged with the 3xFLAG-tag was used as a positive control for enChIP

The orthologues of Cas9 can be used for sequential enChIP, which might be useful for decreasing the background noise of either system alone. However, for sequential enChIP, CRISPR complexes of different species must be pulled down using different affinity-purification systems (e.g., purification using Abs against each dCas9 or different epitope tags). To this end, we generated a lentiviral expression system of *S. pyogenes* CRISPR in which Sp-dCas9 was fused with an NLS and the 2xAM-tag (Sp-dCas9-2xAM) (Fig. 2a). The lentiviral expression system allows quick establishment of stable transformants expressing the CRISPR complex even when the target cells are not proliferating. The coding region of Sp-dCas9-2xAM was inserted into the CSII-U6-gRNA-CBh-3xFLAG-PA-dCas9-P2A-Puro plasmid to generate pLenti_dCas9-2xAM. The gRNA dsDNA targeting the *IRF*-*1* promoter was inserted into pLenti_dCas9-2xAM to generate pLenti_dCas9-2xAM_hIRF-1.

To confirm that the system (Fig. 2b) works, pLenti_dCas9-2xAM or pLenti_dCas9-2xAM_hIRF-1 was transduced into a human fibrosarcoma cell line, HT1080. After puromycin selection, expression of Sp-dCas9-2xAM was confirmed by immunoblot analysis with Ab against the AM-tag (Fig. 2c).

Next, we examined yields of enChIP for the target *IRF*-*1* promoter region. Cells expressing Sp-dCas9-2xAM in the absence or presence of gRNA targeting the *IRF*-*1* promoter were crosslinked with formaldehyde. The crosslinked chromatin was fragmented by sonication, and complexes containing the CRISPR complexes were subjected to affinity purification using Ab against the AM-tag. The yields of enChIP in the presence of the gRNA were comparable with those obtained using HT1080 expressing 3xFLAG-dCas9 and gRNA targeting the *IRF*-*1* promoter region [16] (Fig. 2d). These results showed that enChIP using the 2xAM-tag can also specifically and efficiently isolate target genomic regions. Because lentivirus can infect non-dividing cells, this enChIP system would also be useful for cells that are not proliferating.

We have not generated stable cell lines expressing Sa-dCas9-3xFLAG. Therefore, at this stage, we cannot compare the enChIP efficiencies between Sa-dCas9-3xFLAG and the lentiviral system of Sp-dCas9-2xAM. However, considering that the enChIP efficiencies between 3xFLAG-Sp-dCas9 and Sa-dCas9-3xFLAG using transient expression systems are comparable (Fig. 1e), and the enChIP efficiencies between 3xFLAG-Sp-dCas9 and Sp-dCas9-2xAM using stable transformants are also comparable (Fig. 2d), we speculate that the enChIP efficiencies between Sa-dCas9-3xFLAG and Sp-dCas9-2xAM might also be comparable.

## Conclusions

In this study, we developed an enChIP system using *S. aureus* CRISPR. Due to its distinct PAM sequence (5′-NNGRRT-3′ or 5′-NNGRR(N)-3′), this system will increase the flexibility of the enChIP technology by increasing the proportion of genomic regions that can be targeted. In addition, we developed another enChIP system using *S. pyogenes* CRISPR fused with a different epitope tag different than the one used for *S. aureus* CRISPR. Both enChIP systems achieved high yields of the target locus, and it is expected that their yields would be comparable.

## Limitations

Further studies might be necessary to assess the utility of the *S. aureus* system in enChIP combined with MS and NGS in order to allow comparisons with features of the *S. pyogenes* system such as background signals, off-target binding, etc. In addition, it would be of interest to determine whether sequential enChIP analysis could be performed using the *S. pyogenes* and *S. aureus* CRISPR systems.

## Abbreviations

enChIP: engineered DNA-binding molecule-mediated chromatin immunoprecipitation
CRISPR: clustered regularly interspaced short palindromic repeats
dCas9: a catalytically inactive form of Cas9
NLS: nuclear localization signal
gRNA: guide RNA
TAL: transcription activator-like
Ab: antibody
MS: mass spectrometry
NGS: next-generation sequencing
PAM: protospacer adjacent motif
NE: nuclear extracts
IRF-1: interferon regulatory factor-1
